# Clinical significance of monocyte heterogeneity

**DOI:** 10.1186/s40169-014-0040-3

**Published:** 2015-02-14

**Authors:** Brian K Stansfield, David A Ingram

**Affiliations:** Department of Pediatrics and Neonatal-Perinatal Medicine, Georgia Regents University, Augusta, Georgia; Vascular Biology Center, Georgia Regents University, Augusta, Georgia; Herman B. Wells Center for Pediatric Research, Georgia Regents University, Augusta, Georgia; Department of Pediatrics and Neonatal-Perinatal Medicine, Indiana University School of Medicine, Indianapolis, Indiana USA; Department of Biochemistry and Molecular Biology, Indiana University School of Medicine, 699 Riley Hospital Drive, RR208, Indianapolis, IN 46202 USA; Medical College of Georgia at Georgia Regents University, 1120 15th St, BIW-6033, Augusta, GA 30912 USA

**Keywords:** Monocyte, CD14, CD16, Ly6C, Macrophage, Cardiovascular, Atherosclerosis, Autoimmune Disease, Human, Mouse

## Abstract

Monocytes are primitive hematopoietic cells that primarily arise from the bone marrow, circulate in the peripheral blood and give rise to differentiated macrophages. Over the past two decades, considerable attention to monocyte diversity and macrophage polarization has provided contextual clues into the role of myelomonocytic derivatives in human disease. Until recently, human monocytes were subdivided based on expression of the surface marker CD16. “Classical” monocytes express surface markers denoted as CD14^++^CD16^−^ and account for greater than 70% of total monocyte count, while “non-classical” monocytes express the CD16 antigen with low CD14 expression (CD14^+^CD16^++^). However, recognition of an intermediate population identified as CD14^++^CD16^+^ supports the new paradigm that monocytes are a true heterogeneous population and careful identification of specific subpopulations is necessary for understanding monocyte function in human disease. Comparative studies of monocytes in mice have yielded more dichotomous results based on expression of the Ly6C antigen. In this review, we will discuss the use of monocyte subpopulations as biomarkers of human disease and summarize correlative studies in mice that may yield significant insight into the contribution of each subset to disease pathogenesis.

## Introduction

**Figure 1:**
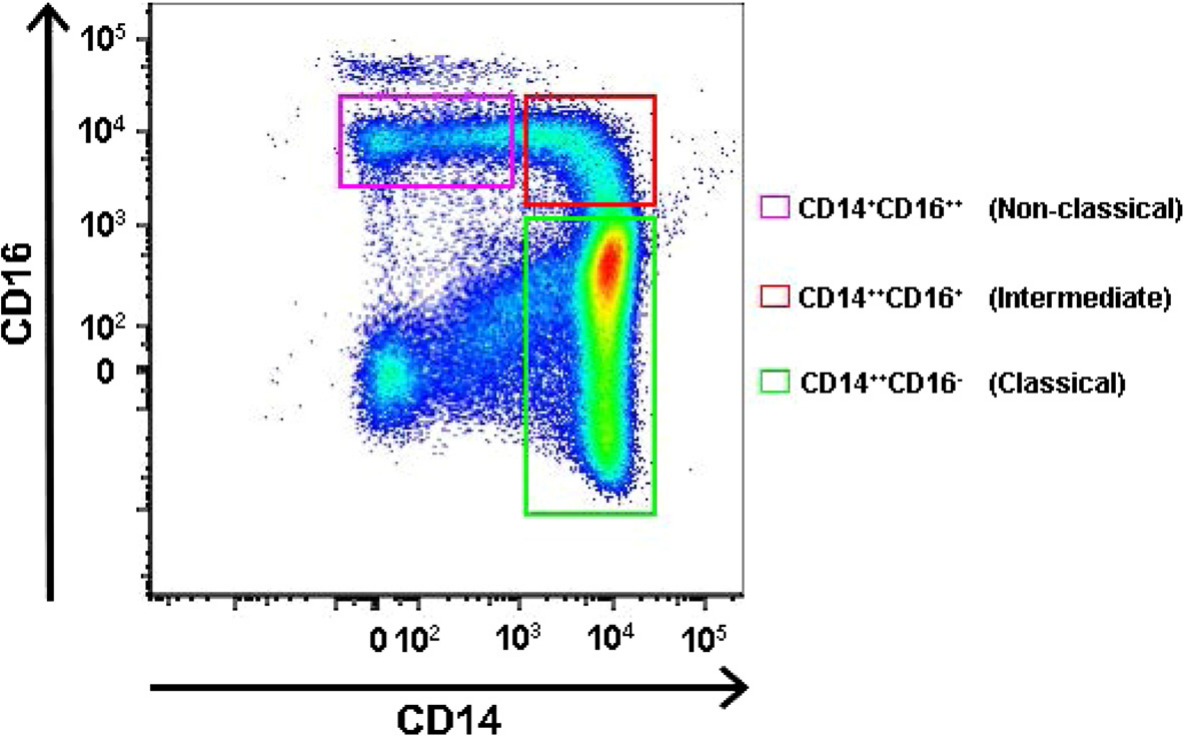
**Sample gating strategy showing three human monocyte populations based on expression of CD14 and CD16.** Relative expression of CD14 (x-axis) and CD16 (y-axis) is demonstrated on log scale. Classical monocytes (CD14^++^CD16^−^) are gated in the green box, intermediate monocytes (CD14^++^CD16^+^) in the red box, and non-classical monocytes (CD14^+^CD16^++^) in the lavender box.

Until the late 1980s, monocytes were considered to represent a single population of circulating hematopoietic cells derived from the common myeloid progenitor cell in the bone marrow. The seminal work of Passlik et al. demonstrated that distinct monocyte subsets could be identified based on the expression of the surface antigen CD16 [1]. “Classical” monocytes do not express the CD16 antigen (CD14^++^CD16^−^), while “non-classical” monocytes are smaller in size and express CD16 on the cell surface (CD14^+^CD16^++^) [1]. The expression pattern of the two surface markers lends some insight into their function. CD14 acts as a co-receptor for toll-like receptor 4 and mediates lipopolysaccharide (LPS) signaling, while the CD16 antigen is identified as FcγRIIIa and participates in innate immunity [2,3]. The subsequent two decades have yielded considerable insight into the role of each cell population in human disease; however, the recent emergence of an intermediate monocyte population denoted as CD14^++^CD16^+^ has shifted focus away from this simple classification system [4-8] (Figure 1). As of 2010, three distinct monocyte populations outlined by Ziegler-Heitbrock et al. are officially recognized: CD14^++^CD16^−^ (classical), CD14^++^CD16^+^ (intermediate), and CD14^+^CD16^++^ (non-classical) [4]. We will adhere to the official nomenclature for this review article.

Classical monocytes represent the largest population of monocytes and are important scavenger cells [4]. Non-classical monocytes were often referred to as pro-inflammatory, secondary to their mobilization in disease and secretion of important inflammatory cytokines [7,9-11] (i.e. TNF-α). The new tripartite view of monocyte subpopulations has shifted attention from the inflammatory characteristics of non-classical monocytes and supports a more significant role for intermediate monocytes in inflammation. Further, unique functions in angiogenesis, production of reactive oxygen species, and patrolling behavior have been attributed to CD16^+^ monocytes [12-18]. In the present review, we will summarize the contribution of monocyte heterogeneity in human disease and draw across multiple common pathologies to characterize each monocyte subtype for clinical relevance. We will focus on the impact of monocytes in cardiovascular disease, including myocardial infarction, atherosclerosis, and stroke, chronic kidney disease, and autoimmune disease. Finally, we will identify distinct murine monocyte populations and link their function in preclinical animal models to human monocyte function.

## Review

### Monocyte ontogeny and heterogeneity

Genetic profiles, antigen expression, and cytokine production of each monocyte population provide a foundation for hierarchical clustering and classification. Cros et al. showed that CD14^+^CD16^++^ monocytes (non-classical) are segregated independently from intermediate and classical monocytes based on principal component analysis [14]. Functionally, CD14^++^CD16^−^ and CD14^++^CD16^+^ monocytes are more phagocytic than non-classical monocyte [14]. Conversely, other investigators have provided genetic and functional evidence to suggest that CD16^+^ monocytes cluster together and are more distinct from classical CD16^−^ monocytes [7,16,19-21]. Wong et al. showed significantly different genetic profiles between intermediate and classical monocytes, which supported independent findings in humans and rhesus monkeys [13,19,22]. Interestingly, cytokine production in response to LPS *in vitro* reveals a more complex relationship. Intermediate monocytes produced significantly more tumor necrosis factor-α (TNF-α) and interleukin-1β (IL-1β) compared to the other populations; however, production of interleukins 6, 8, and 10 were approximate in intermediate and classical monocytes when compared to non-classical monocytes [14,19].

Despite this controversy, stereotypic functions of classical and non-classical monocytes are beginning to emerge. CD14 is a pattern recognition receptor and classical monocytes are critical components of innate immunity, while CD16^+^ (intermediate and non-classical) monocytes are preferentially mobilized during inflammation and lend to their designation as “pro-inflammatory” [16,23,24]. The significant functional overlap of intermediate monocytes with classical and non-classical monocytes lends credence to the present hypothesis that CD14^++^CD16^−^ monocytes arise from the bone marrow and give rise to CD14^++^CD16^+^ and CD14^+^CD16^++^ monocytes [4,13,24]. CD14^++^CD16^−^ monocytes primarily produce monocyte chemotactic protein-1 (MCP-1/CCL2) and express its cognate receptor CCR2, as do intermediate monocytes [14,22]. CCR2 is critical for monocyte emigration from the bone marrow and monocyte homing [25-29]. Likewise, CD62L (SELL), expressed on classical monocytes, is an important early marker of progenitor cell commitment to specific progeny [30,31]. Population specific genetic profiles reveal a progressive increase in genes associated with maturation in intermediate and non-classical monocytes when compared with classical monocytes [19,24]. Finally, in response to macrophage colony stimulating factor (M-CSF), intermediate monocytes are initially expanded in the peripheral blood followed by a delayed increase in non-classical monocyte frequency [24,32,33]. Though not dogmatic, general recognition that classical monocytes arise from the bone marrow and give rise to intermediate and non-classical monocytes has provided a platform for understanding monocyte maturation and methods of replenishment in disease states. Table 1 outlines the expression of surface markers for classical, intermediate, and non-classical monocytes.

**Table 1:** **Profile of human and mouse monocyte subpopulations**

	**Human**			**Mouse**	
	**CD14** ^**++**^ **CD16** ^**−**^	**CD14** ^**++**^ **CD16** ^**+**^	**CD14** ^**+**^ **CD16** ^**++**^	**Ly6C** ^**high**^	**Ly6C** ^**low**^
CCR2^a,b,c^	++	+	-	++	+
CX_3_CR1^a,b,c^	+	++	+++	+	++
CD62L^a,c^	++	+/−	-	++	-
CXCR1^a,c^	++	+/−	-	ND	ND
CXCR2^a,c^	++	+/−	-	ND	ND
PSGL-1^d^	++	+	+	++	+
CD11a^e,f^	+	+	++	+	++
CCR5^a,b,c^	+	++	-	+	+
ACE^g^	+	++	+	ND	ND
HLA-DR^a^	-	++	+	ND	ND
CD11b^a^	+	+	+/−	+	+
CD11c^e,h,i^	-	+	+	-	+
CD43^f^	-	+	++	+	++
MHCII^a,e,h^	+	++	+	-	+

^a^Wong et al. Blood 2011; ^b^Rogacev et al. Eur Heart J 2011; ^c^Ancuta et al. JEM 2003; ^d^An et al. Circulation 2008; ^e^Ingersoll et al. Blood 2009; ^f^Zawada et al. Blood 2011; ^g^Ulrich et al. Nephrol Dial Transplant 2010; ^h^Sunderkotter et al. J Immunol 2004; ^i^Tanaka et al. Clin Exp Immunol 1999.

Presently, murine monocytes are evenly distributed by their relative expression of the Ly6C antigen and are, for the most part, functionally distinct [34-39]. Ly6C^high^ monocytes co-express CCR2 and CD62L with low expression of the fractalkine receptor CX_3_CR1, whereas Ly6C^low^ monocytes have elevated expression of CX_3_CR1 with lower expression of CCR2 [39,40]. This expression pattern supports the present view that Ly6C^high^ monocytes are closely related to human classical (CD14^++^CD16^−^) monocytes and conversely, Ly6C^low^ monocytes are analogous to the non-classical CD14^+^CD16^++^ population [14,19,24,40,41]. Bone marrow cells are enriched for the Ly6C antigen and Ly6C^high^ monocytes are capable of giving rise to Ly6C^low^ monocytes and other derivatives in some animal models [35,42-46]. However, Ly6C^high^ monocytes are preferentially recruited in inflammation and are the major source of cytokines such as TNF-α and IL-1β [36,37,40]. This departure from the functional description of classical human monocytes requires resolution and may be a function of current analytical techniques. For example, separation techniques leverage the expression of human monocyte markers to label each monocyte with differentially expressing antibodies. The binding of these labeling antibodies to poorly expressed surface antigens may preclude their activation and underestimate cytokine production and their response to various stimuli including LPS [16]. Additionally, expression patterns of classic markers for human monocytes (CD14, CD16, CD86, etc.) and murine markers (Ly6C, CCR2, CX3CR1) are not directly analogous between species. CCR2, for example, is preferentially expressed on Ly6C^high^ murine monocytes and has provided evidence for their functional similarity to classical human monocytes. However, CCR2 is expressed on Ly6C^low^ monocytes as well, but is poorly expressed on human CD16^+^ monocytes [41]. Further, these simple murine monocyte profiles (inflammatory vs. pro-inflammatory) likely limit our ability to understand individual murine monocyte function and hinder translation to human studies. Ingersoll et al. compared human CD16- and CD16+ monocytes with their murine counterpart to show that multiple pro-inflammatory and phagocytic antigens (TREM-1, CD64 and CD36) were highly expressed on Ly6C^low^ monocytes and approximated relative expression in CD16- human monocytes [41]. However, antigen expression patterns and genomic data mostly confirm the present grouping of Ly6C^high^ monocytes with CD14^++^CD16^−^ human monocytes [14,40,41]. Several recent reviews attempt to resolve this controversy [24,39,40].

### Monocytes phenotypes in cardiovascular disease

Expansive remodeling and leukocyte infiltration of the left ventricle in response to coronary artery occlusion is predictive of poor outcomes [40,47]. Monocyte mobilization during the evolving infarction and recovery period follow a sequential pattern. Tsujioka et al. studied the mobilization of CD16^−^ and CD16^+^ monocytes during the natural progression of acute myocardial infarction (AMI) in a Japanese patient population [48]. Circulating CD16^+^ monocytes were diminished in patients diagnosed with AMI at the time of admission and rose significantly over the first week after diagnosis. The mobilization of CD16^+^ monocytes was preceded by increased CD14^++^CD16^−^ monocytes in the first days following AMI admission. Circulating monocyte populations did not differ between groups; however, peak levels of CD14^++^CD16^−^ monocytes were a negative predictor of myocardial salvage and no relationship between CD16^+^ monocytes and infarction size was noted in this study [48]. Other human studies and murine models of myocardial infarction support these findings [50-53]. Ly6C^high^ monocytes, corresponding to classical human monocytes, accumulate in the myocardium immediately after AMI and are followed by a recovery period denoted by expansion of Ly6C^low^ monocytes (analogous to human non-classical monocytes) [50,54]. However, characterization of human monocyte mobilization in Tsujioka et al. is difficult to interpret, as CD16^+^ monocytes were not distinguished into intermediate and non-classical populations [48]. Recently, monocyte subtypes were analyzed in a cohort of patients admitted for AMI and compared to patients with stable coronary artery disease (CAD) and a second cohort of healthy controls [55]. Patients admitted for AMI had a 60-80% increase in total monocyte count and a disproportionate increase (3-fold) in intermediate (CD14^++^CD16^+^) monocytes when compared with patients admitted with CAD and healthy controls. The intermediate monocyte population remained elevated for the subsequent 7 days after AMI and trended to background control levels by day 30. The authors go on to show that return of the intermediate monocyte population to normal frequency correlated with decreased troponin levels and return of left ventricular ejection fraction [55]. These smaller cohort studies are supported by a large prospective cohort study of persons undergoing angiography. Intermediate monocyte frequency strongly correlated with a composite outcome of cardiovascular death, non-fatal AMI, and non-hemorrhagic stroke in 951 patients. A planned secondary analysis revealed that patients with the highest frequency of intermediate monocytes had a 40% risk of experiencing a cardiovascular event within three years of study enrollment [49]. Collectively, clinical studies suggest an intriguing role for monocyte differentiation as a diagnostic and predictive biomarker in patients suffering AMI.

Similar to the findings observed in patients suffering AMI, a characterization study of human monocyte mobilization in the days following a stroke revealed an expansion of intermediate monocytes by day 2, while the classical monocyte population was unperturbed [56]. Non-classical CD14^+^CD16^++^ monocyte frequency inversely mirrored intermediate monocyte frequency and reached the nadir by day 2 after stroke. The classical monocyte population was lower at the time of admission for stroke in surviving patients and correlated with improved prognosis. Intermediate monocyte mobilization over the subsequent 48 hours following stroke also correlated with stroke severity and increased mortality rates, which held significance following adjustment for age and stroke severity scoring [56,57]. A second cohort of 36 stroke patients supported the increased frequency of intermediate monocytes in the peripheral blood in the days to weeks following infarction; however, Kaito et al. showed that classical monocyte numbers increased immediately following presentation for stroke [58]. A positive correlation with stroke severity score in this study was also observed and may be explained by a strong association with progressing infarction [58]. Thus, assessment of monocyte subsets following admission for stroke may show a time-dependent predictive value in outcomes and survival.

### Monocytes in chronic cardiovascular inflammation

Atherosclerosis is the result of complex interactions between circulating leukocytes and the vascular wall, which are likely propagated by chronic secretion of growth factors and inflammatory cytokines. The lack of true homology between murine and human monocyte subpopulations limit the direct application of findings in murine atheroma to that observed in human cohorts. Ly6C^high^ monocytes, the murine correlate of human classical monocytes, drive atherosclerosis in transgenic animal models based on the genetic deletion of apolipoprotein E or the LDL receptor [26,37,45,59-61]. Ly6C^high^ monocytes preferentially bind to the activated endothelium in atherogenic ApoE knockout mice and infiltrate the developing atheroma to give rise to lesional macrophages and, eventually, foam cells [26,37]. Comparatively, CD16^+^ monocytes are primarily implicated in human atherosclerosis. Increased frequency of non-classical monocytes, in association with other markers of dyslipidemia, has been demonstrated in multiple human studies [24,62,63]. Non-classical monocytes are mobilized in patients with hypercholesterolemia and their frequency in the peripheral blood appears to be directly correlated with total cholesterol and triglyceride level and inversely proportional to HDL concentration [62-64]. Recent work attempting to differentiate the contribution of CD16+ monocytes to atherosclerosis show that intermediate monocyte frequency is closely related to severity of angina and may contribute to atherosclerosis [65,66]. These findings have implications on therapeutic efficacy since monocyte subpopulations have variable expression of scavenger receptors that participate in cholesterol uptake and metabolism. For instance, an early randomized trial targeting hypercholesterolemia showed that statin treatment (fluvastatin) increased CD16^+^ monocytes [63]. Other studies have not identified a similar increase in non-classical monocytes following initiation of a statin (rosuvastatin, atorvastatin, and pivastatin), which may suggest class-dependent effects on monocyte frequency [67,68].

To compound the difficulty of interpreting these studies, the vast majority of human studies failed to include the intermediate monocyte population in their analysis. Recently, intermediate monocyte frequency was identified as a positive predictor of major cardiovascular event in two large European cohorts of 438 and 951 patients [49,69]. Further, intermediate monocyte frequency negatively correlated with HDL and Apo-1 [49,69], which is likely consistent with previous clinical studies demonstrating an association between CD16^+^ monocytes and markers of dyslipidemia. These findings are particularly intriguing since intermediate monocytes may possess significant inflammatory properties and exaggerate oxidative stress when compared with non-classical monocytes. Intermediate monocytes express CCR2 and CCR5, which are critical for monocyte homing and trans-endothelial migration into the atherosclerotic plaque [26,27,61,70]. Genetic deletion of CCR2 or CCR5 in ApoE knockout mice significantly reduces atherosclerosis and monocyte mobilization [71-75]. Further exploration of the relationship between intermediate (CD14^++^CD16^+^) monocytes and atherosclerosis will significantly advance our understanding of pro-atherogenic conditions and aide in the design of novel cell-directed therapies for the treatment of atherosclerosis.

Obesity represents a chronic inflammatory state that increases the risk for heart disease and stroke by greater than fifty percent [76,77]. Several groups have independently demonstrated that the frequency of non-classical and intermediate monocytes positively correlates with WHO obesity classification and fat mass [24,64,78-80]. In fact, a cohort of 105 subjects with WHO class II and III obesity had an increased frequency of non-classical and intermediate monocytes in the peripheral blood compared to healthy controls; a finding that was not observed in WHO class I subjects [64]. A modest 5% reduction in fat mass significantly reduced non-classical monocyte frequency and improved glycemic control in obese diabetic patients [64]. Not surprisingly, the vast majority of positive correlative studies between non-classical monocyte frequency and cardiovascular disease loose statistical significance in post-hoc analysis correcting for BMI. These epidemiologic observations in human cohorts provide evidence for a direct link between adiposity, inflammation, and monocyte maturation.

Animal models of metabolic disease may provide some clues to the connection between obesity and vascular inflammation. ApoE knockout (ApoE^−/−^) mice provided a high fat western diet develop morbid obesity and have evidence of chronic inflammation. Loss of ApoE expression in hematopoietic cells appears to drive monocyte proliferation, mobilization, and differentiation in the development of atherosclerosis [81]. ApoE^−/−^ mice on high fat diet (HFD) developed a time-dependent exponential increase in Ly6C^high^ monocyte frequency in the peripheral blood and spleen compared to wildtype mice on HFD [37]. Ly6C^high^ monocytes rapidly incorporated into the developing atheroma and accounted for the vast majority of lesional macrophages [37,45]. However, local macrophage turnover within atheroma may drive foam cell formation independent of monocyte recruitment [36]. Several studies have demonstrated that ApoE interacts with the ATP-binding cassette transporters (i.e. ABCA1) to coordinate cholesterol efflux and monocyte recruitment [81-83]. Loss of ABCA1, and other cholesterol transport proteins, accelerates lesion formation and likely participates in myeloid cell proliferation and function.

Recent attention to the role of adventitial adipose tissue, termed perivascular adipose tissue or PVAT, provides a novel look at the relationship between adiposity and vascular inflammation. Perivascular adipocytes secrete growth factors and adipokines that stimulate an inflammatory response by recruiting monocytes and other leukocytes to the vascular wall [84-88]. Precursor and differentiated adipocytes accumulate near atheroma in the perivascular adventitia of conduit vessels and expand with obesity [86]. Monocyte chemotactic protein-1 (MCP-1/CCL2) is highly secreted by perivascular adipocytes and is a potent mitogen for circulating monocytes [85,89]. Genetic deletion of MCP-1 in mice impedes macrophage infiltration and lipid deposition following arterial injury [85]. Interestingly, MCP-1 is sequentially expressed in the adventitia followed by the media and intima after coronary artery injury, suggesting a central role for perivascular adipocytes in monocyte recruitment [90]. These data provide evidence that perivascular adiposity, along with visceral and subcutaneous fat, participate in vascular inflammation and monocyte chemotaxis. How perivascular adipocytes interact with circulating hematopoietic cells and isolating their effect on leukocyte recruitment from other fat stores in vascular inflammation are important future areas of investigation.

### Monocyte subpopulations in kidney disease

Chronic kidney disease (CKD) is closely related with cardiovascular health and commonly exists as a co-morbid condition in persons with cardiovascular disease. Persons with CKD often suffer from atherosclerosis and are at increased risk for mortality following an acute cardiovascular event [91,92]. Patient cohorts have uniformly demonstrated that increased intermediate (CD14^++^CD16^+^) monocyte frequency is associated with cardiovascular event rate and increased mortality [70,78,93-95]. A study of 119 patients with CKD demonstrated an increased frequency of intermediate monocytes in the peripheral blood of hemodialysis-dependent CKD patients compared to CKD patients with adequate native renal function [70]. Depletion of intermediate monocytes during hemodialysis was associated with an increased cardiovascular event-free period and reduced mortality in patients with end-stage renal disease [70,96]. Classical and non-classical monocytes have failed to show a correlation with mortality in CKD patients regardless of the need for hemodialysis. Subgroup analysis identified intermediate monocytes expressing angiotensin-converting enzyme (ACE) were significantly higher in CKD patients with severe atherosclerosis and may yield additional prognostic value [94-96]. Increased ACE expression in intermediate monocytes has also been demonstrated in patients with abdominal aortic aneurysm and appeared to be independent of CKD classification [97]. While the role of ACE (CD143) on circulating monocytes remains unknown, the renin-angiotensin system is critical for monocyte maturation and function and targeting this signaling axis may be of therapeutic benefit in monocyte-driven human disease [98,99]. Along this line of inquiry, a small cohort of dialysis-dependent CKD patients was randomized to an angiotensin receptor 1 blocker (losartan) or other anti-hypertensive medication and monocyte populations were evaluated. The losartan group showed a reduction of CD16^+^ monocytes in their peripheral blood after two months of therapy compared to patients receiving antihypertensive medication that did not target the renin-angiotensin axis [100]. The authors demonstrate that the CD16^+^ monocytes produced TNF-α *in vitro*; however their gating strategy failed to distinguish intermediate and non-classical monocytes within the CD16^+^ population. While CD14^++^CD16^+^ monocytes appear to be an important biomarker of CKD severity, their role in the evolution of CKD is yet to be determined.

### Monocytes and autoimmune disease

Monocyte-mediated inflammation has long been targeted in the treatment of rheumatoid arthritis. Attention to monocyte heterogeneity has expanded our understanding of rheumatologic diseases and provided new interventions for patients. Most groups have demonstrated an expansion of CD16^+^ monocytes in patients with rheumatoid arthritis with the recent delineation of CD16^+^ subpopulations strongly suggesting that CD14^++^CD16^+^ monocytes are the principle inflammatory effectors [21,101,102]. CD16^+^ monocytes are found in the synovial fluid of RA patients and are associated with joint destruction [101]. Elevation of the CD16^+^ monocyte frequency in RA patients was recently demonstrated to be primarily an expansion of the intermediate population [21]. Sequestering the inflammatory properties of CD14^++^CD16^+^ monocytes may inhibit joint injury and signify response to therapy. Monoclonal antibodies directed against TNF-α (Inflixamab, Adalimumab, Etanercept) have been extremely effective in the treatment of RA and may target, in part, intermediate and non-classical CD16^+^ monocytes. A comparison study of anti-TNF-α antibodies demonstrated a reduction in CD14^++^HLADR^+^ monocytes in RA patients taking infliximab that the authors suggest may account for the increased sepsis event rate noted in this study population [103]. HLADR is an important third marker (along with CD86) that is highly expressed on intermediate monocytes and less so on non-classical monocytes [104]. A larger cohort of RA patients (111 total) showed a slight, but non-significant increase in intermediate monocyte frequency in early RA that was profoundly increased in RA patients with long-standing disease [105]. In response to treatment with methotrexate, the authors identified that CD16 expression on CD14^++^ monocytes was a negative predictor of response after 14 weeks of therapy. Both RA patients with and without a history of treatment with immune modulating agents showed a negative correlation between intermediate monocyte frequency during treatment and disease activity scores [105]. These clinical studies suggest that intermediate monocyte frequency may predict response to therapy and side effect profile in RA patients using immunosuppressive therapy. However, cytokine production has not been linked to monocyte subpopulation frequency in RA patients during active disease or following treatment. Therefore, the use of monocyte markers as a biomarker for therapeutic response remains limited.

Similar to RA patients, CD16^+^ monocytes are preferentially expanded in persons with both Crohn’s Disease (CD) and ulcerative colitis [11,106,107]. Grip et al. showed a spike in intermediate monocyte frequency in CD patients with active disease, which was marked by increased expression of CCR2 on the intermediate monocyte population. Classical and intermediate monocytes expressed similar levels of CCR2 during active disease and the expression pattern of CCR2 on the intermediate monocyte population in CD patients was significantly elevated compared to the expression of CCR2 on matched healthy controls [11]. These findings are intriguing and suggest that monocytes, within a sub-classification system, may have alternative phenotypic signatures in response to inflammation. Further, this data supports the independent finding that MCP-1 (CCL2) levels are increased in patients with inflammatory bowel disease and correlate with disease severity [108,109]. Recently, Thiesen et al. showed enhanced migration of classical monocytes isolated from CD patients in response to MCP-1 (CCL2) and increased CD14 expression in macrophages isolated from inflamed intestinal mucosa of CD patients [110]. The CD14^++^ HLADR^low^ macrophages isolated from the colon of CD patients expressed high levels of CD62L which supports a classical monocyte origin, however, these macrophages also expressed the fractalkine receptor CX_3_CR1 which is more characteristic of non-classical monocytes [110]. Other groups have found a more pronounced infiltration of CD16^+^ monocytes in actively inflamed tissue from CD patients that corresponded with increased staining for TNF-α [107]. Resolution between these findings may again point back to an integral role for intermediate monocytes in active CD.

### Gaps and future directions in understanding monocyte heterogeneity

Recent adherence to the tripartite description of monocytes based on expression of CD14 and CD16 along with standardized gating strategies has enabled investigators to more thoroughly explore the nature of monocyte differentiation and function in human disease. However, the recognition of an intermediate population that most truly represents an inflammatory monocyte casts a shadow on previous studies demonstrating expansion of classical and non-classical monocytes during inflammation. Thus, a re-examination of previous clinical studies and human cohorts with a uniform gating strategy and consistent use of approved nomenclature is warranted.

Though evidence of monocyte mobilization in human disease has now been demonstrated, the question of whether their rise is consequential of the illness/inflammation or directly contributes to the inflammatory process remains largely unanswered. In particular, does the increase in CD16^+^ monocyte (intermediate and non-classical) frequency observed in cases of both acute and chronic inflammation result from increased differentiation or do they arise independently in response to inflammatory signaling cascades. Human studies addressing these questions will likely focus on chronic diseases with periodic episodes of acute inflammation (i.e. autoimmunity), as several groups have pointed out that classical monocytes appear to exhibit a progressive increase in CD16 expression during acute illness [107,111,112]. A particularly interesting subset of these patients includes those with disease primarily affecting a single organ such as RA or Crohn’s Disease. Comparative studies of monocyte frequency between the peripheral blood and diseased organ have generated promising data supporting the maturation of these monocytes in the peripheral blood prior to infiltration. Further, does the spleen function as a monocyte reservoir for mobilization during periods of acute inflammation as has been observed in mice [45]? Supportive *in vitro* characterization of all monocyte populations, especially the intermediate subset, will help define the inflammatory signature of each population and may lead to the designation of a true pro-inflammatory monocyte. Specific therapeutic targeting of the offending monocyte population may limit the need for treatments that indiscriminately target all monocytes and the important side effect profile associated with these types of therapies. Finally, could preferential recruitment of a less inflammatory monocyte population limit inflammation and promote healthy repair?

## Conclusions

Recognition of three monocyte populations with diversified and heterogeneous responses in health and disease has increased mechanistic insight into the pathogenesis of inflammation as it relates to both chronic and acute illness. The more dichotomous murine monocyte populations, denoted as Ly6C^high^ and Ly6C^low^, hinder the ability to directly translate animal findings to humans. Careful mechanistic and lineage tracing studies in mice and humans will enhance our understanding of monocyte function in health and disease. At present, the intermediate monocyte population appears to represent an inflammatory monocyte population and therefore harnessing the inflammatory capacity of intermediate monocytes may promote a healthy response to injury or inflammation. Selective depletion of intermediate monocytes requires a highly specific surface marker regimen. Therefore, uniform adoption of the recommended gating strategies and nomenclature for proper identification of each monocyte subpopulation is necessary to provide a clear picture of their role in human disease [4,5,24].

## Abbreviations

ABCA1: ATP-binding cassette transporter-1
ACE: Angiotensin-converting enzyme
AMI: Acute myocardial infarction
Apo-1: Apolipoprotein-1
ApoE: Apolipoprotein-E
CAD: Coronary artery disease
CCR2: C-C motif receptor-2
CCR5: C-C motif receptor-5
CD: Crohn’s disease
CD14: Cluster of differentiation 14
CD16: Cluster of differentiation 16
CD62L/SELL: Cluster of differentiation 62 L
CD86: Cluster of differentiation 86
CKD: Chronic kidney disease
CX_3_CR1: Fractalkine receptor
HDL: High density lipoprotein
HFD: High fat diet
HLADR: Human leukocyte antigen-DR
IL-1β: Interleukin-1β
LDL: Low density lipoprotein receptor
LPS: Lipopolysaccharide
Ly6C: Lymphocyte antigen 6C
MCP-1/CCL2: Monocyte chemotactic protein-1/ chemokine (C-C Motif) ligand-2
M-CSF: Macrophage-colony stimulating factor
PVAT: Perivascular adipose tissue
RA: Rheumatoid arthritis
TNF-α: Tumor necrosis factor-α
